# Estimated impact of the COVID-19 pandemic on the prevalence and treatment of depressive symptoms in Peru: an interrupted time series analysis in 2014–2021

**DOI:** 10.1007/s00127-023-02446-8

**Published:** 2023-03-08

**Authors:** David Villarreal-Zegarra, C. Mahony Reátegui-Rivera, Sharlyn Otazú-Alfaro, Gloria Yantas-Alcantara, Percy Soto-Becerra, G. J. Melendez-Torres

**Affiliations:** 1 Escuela de Medicina, Universidad César Vallejo, Trujillo, Peru; 2 Instituto Peruano de Orientación Psicológica, Lima, Peru; 3 Unidad de Telesalud, Universidad Nacional Mayor de San Marcos, Lima, Peru; 4 Universidad Continental, Huancayo, Perú; 5 College of Medicine and Health, University of Exeter, Exeter, UK

**Keywords:** Depressive symptoms, Prevalence, Treatments, Epidemiology, Public health

## Abstract

**Purpose:**

The COVID-19 pandemic increased the burden of mental disorders worldwide. Peru has been one of the countries most affected by COVID-19, however, studies evaluating the medium and long-term consequences of the pandemic on Peruvians’ mental health are recent and represent a new field of study in proliferation. We aimed to estimate the impact of the COVID-19 pandemic on the prevalence and treatment of depressive symptoms using nationally representative surveys in Peru.

**Methods:**

Our study is an analysis of secondary data. We carried out a time series cross-sectional analysis based on the National Demographic and Health Survey of Peru, collected using a complex sampling design. The Patient Health Questionnaire-9 was used to measure mild (5–9 points), moderate (10–14 points), and severe (15 points or more) depressive symptoms. The participants were men and women aged 15 years and older, living in urban and rural areas of all regions of Peru. The main statistical analysis used segmented regression with Newey-West standard errors, taking into account that each year of the evaluation was divided into four measures (quarter measure).

**Results:**

We included 259,516 participants. An average quarterly increase of 0.17% (95% CI 0.03–0.32%) in the prevalence of moderate depressive symptoms was identified after the onset of the COVID-19 pandemic (approximately an increase of 1583 new cases of moderate depressive symptoms by each quarter). The percentage of cases treated for mild depressive symptoms increased quarterly by an average of 0.46% (95% CI 0.20–0.71%) after the onset of the COVID-19 pandemic (approximately an increase of 1242 new cases treated for mild depressive symptoms by each quarter).

**Conclusion:**

In Peru, increases in the prevalence of moderate depressive symptoms and the proportion of cases treated with mild depressive symptoms were found after the COVID-19 pandemic. Therefore, this study is a precedent for future research assessing the prevalence of depressive symptoms and the proportion of cases receiving treatment during the pandemic and post-pandemic years.

**Supplementary Information:**

The online version contains supplementary material available at 10.1007/s00127-023-02446-8.

## Introduction

Prior to the COVID-19 pandemic, depression was among the leading causes of disability-adjusted life year burden worldwide, with an increase of 64% in its prevalence between 1990 and 2019 [1]. In 2020, after the onset of the pandemic, there was an estimated 28% increase in the global prevalence of depression due to the effects of COVID-19 [2]. Meta-analyses suggest that rates of depression in the general population increased approximately sevenfold during the COVID-19 outbreak [3] and in the virus-affected population, the prevalence was threefold compared to the general population [4]. Moreover, access to treatment for depression in low-and-middle-income countries (LMICs) is insufficient and inequitably distributed, with more than 75% of people with depressive symptomatology going untreated [5] and only a third recognizing the need for treatment [6].

Though LMICs are diverse, these countries share similarities in underfinanced health systems, disorganized mental health services, a lack of training and application of evidence-based therapeutic interventions, and the slow implementation of laws and policies that reduce the mental health gap [7]. The result of these deficiencies has repercussions on the health of the population, with a direct relationship between poverty and poor mental health [8].

In Peru, an upper middle-income country, the most recent depressive symptoms prevalence study found no variation in trends of depressive symptomatology and its treatment from 2014 to 2018, but identified urban–rural inequalities in mental healthcare accessibility [9]. Moreover, in 2018, six out of every 100 Peruvians presented moderate to severe depressive symptoms and in line with global trends, women were more likely to have clinically relevant depressive symptoms than men; in the same way people aged 45 years and older, living in the Andean regions and having comorbidities with non-infectious chronic diseases [10]. During the pandemic and the quarantine period in Peru, an increase in depressive symptoms equivalent to five times the national prevalence in 2018 was reported, that is, 3 out of 10 Peruvians had moderate to severe levels of depressive symptoms. People with lower educational level, lower family income and/or unemployed being the most affected [11]. These studies underscore the sociodemographic factors such as being a woman, living in the Andes and having comorbidities contribute to the unequal mental health burdens. However, impacts of the COVID-19 pandemic may be offset by recent health system reforms implemented by the Peruvian government to improve the mental health of its population [12]. Therefore, this study aims to estimate the impact of the COVID-19 pandemic on the prevalence and treatment of depressive symptoms during the COVID-19 pandemic using nationally representative surveys in Peru between 2014 and 2021.

## Methods

### Study design

This interrupted time-series analysis used time-series cross-sectional data from 2014 to 2021 derived from the National Demographic and Health Survey of Peru (DHS-P), a national-level household survey that aims to measure public health indicators [13]. Our study performs a secondary data analysis of the DHS-P. Since 2014, DHS-P has expanded its scope to include a general health assessment (Health Survey) applied to a representative sample of people ages 15 and older [14, 15]. The sampling design and months evaluated are detailed in Supplementary File 1. Until March 2020, data collection was by face-to-face interviews, and the rest of 2020 and 2021 was through a telephone interview with a questionnaire modified due to COVID-19 pandemic.

We included participants aged 15 years or older, living in urban and rural settings in whole Peru, and who have reported complete data on mental health variables (PHQ-9 and mental health treatment) and sociodemographic variables of interest (sex, age, area, economic level, region, and year of evaluation). None exclusion criteria were followed.

The sampling used by DHS-P is probabilistic, two-stage, and representative at both the national and regional levels. The sampling frame for the first stage was the selection of primary sampling units (clusters) based on information from the last Peruvian census. In the second stage, secondary sampling units (households) are selected based on information from cartographic updates and previously conducted housing and building registers.

In rural areas, primary sampling units consist of groups of 500–2000 persons, while secondary sampling units consist of households. In contrast, primary sampling units in urban areas are blocks or groups of blocks, which include more than 2000 persons and an average of 140 households. Secondary sampling units are the same as in rural areas. Refer to the DHS technical documentation for details on the sampling process [14, 15].

Our study used all available secondary data in DHS-P. Because we had no prior estimates of effect (intercept/slope) or within-subject correlations, a power analysis was not possible, however, based on rules of thumb indicating that at least between 8 and 10 Pre and post-intervention measurements [16, 17] are necessary to have adequate power to detect medium-sized effects, the number of time points available for this analysis (32 repeated measurements) is considered sufficient for the purposes of this study.

### Outcomes

The burden of depressive symptoms was measured by PHQ-9. The instrument reports depressive symptomatology in the last two weeks using nine items designed according to the DSM-IV criteria (which remain valid in DSM-V). The scores range from 0 to 27. We used the PHQ-9 severity levels to dichotomize the presence or absence of mild depressive symptoms (scores 0–4); mild depressive symptoms (5–9); moderate depressive symptoms (10–14); and severe depressive symptoms (scores 15 and above) [18, 19]. In a Peruvian representative sample, the PHQ-9 has high reliability (*α* = *ω* = 0.87) and adequate evidence of validity [20]. Our study performed an internal consistency analysis of the PHQ-9 for each of the years evaluated, finding high-reliability values in all years (*α* and *ω* > 0.83).

The percentage of respondents who have received psychological treatment for depression from a health professional in the last year was evaluated. Only participants who responded that they had received psychological treatment and had depressive symptomatology were considered in this study. However, the DHS-P does not collect information on the frequency or type of treatment.

The outcomes were considered continuous because the prevalence and proportion of cases receiving treatment are percentages ranging from 0 to 100%.

### Data analysis

We used interrupted time series, a quasi-experimental approach, to estimate the impact of COVID-19 pandemic on depressive symptoms and treatment prevalence at national level. The intervention period is the pandemic, and the first three months since the pandemic was declared (March to May 2020) were considered censored. This was due to the fact that during those months a strict lockdown was decreed, which made it impossible to collect data from participants. To assess whether the burden of depressive symptoms in the last two weeks and the proportion of people with depressive symptoms receiving treatment changed after the onset of the pandemic, linear regression models compared repeated measures obtained over the entire evaluation time. Segmented regression analysis with Newey-West standard errors was used to model data of measurements before the pandemic (01 January 2014–29 February 2020) and during the lockdown (01 June 2020–31 December 2021). Time units were quarterly and were calculated using the average outcome for that time period. When measurements were not obtained for all months within a quarter (only one or two months), the calculation was made on the basis of the available months within the quarter. The estimated impact of COVID-19 pandemic on outcomes was assessed in terms of level (intercept) change and slope change of the prevalence through the time series before versus after the interruption by the intervention. The change in the intercept is just the immediate change in prevalence level of the outcome, while the change in the slope reflects the change in the trend of prevalence sustained in the time in each quarter. Subgroup analyses were performed for people with lower income levels, living in rural areas, sex, and age group. In addition, for estimates of absolute values, we have assumed that the Peruvian population aged 15 years and older was 24,501,811 people by 2020 [21]. Only complete data were used for analyses. All analyses considered the complex sampling weighting of the DHS-P. Cumby-Huizinga tests (Breusch-Godfrey) with the actest command were used to test for the presence of autocorrelation [22]. Analyses were performed, and tables were created with Stata, version 17.

### Ethics

The DHS-P database used in our study is free and accessible to the general public. The National Institute of Statistics and Informatics (INEI) was responsible for the data collection and the process did not represent an ethical risk for participants. INEI requested the informed consent of participants who were 18 years old and above to obtain the information required in the survey and in the case of minors (17 years old and younger), the request for consent was read to one of their parents or legal guardians to allow the evaluation of the minor.

## Results

### Participants

Initially, 259,645 records were identified. However, after applying the inclusion criteria, a total of 259,516 participants were included in the study (see Table 1). Most of the participants were female (51.7%), and lived in urban areas (75.2%) and in the coastal region of Peru (58.4%). In addition, 61.6% of the participants were married and the mean age was 40.5 years. The characteristics of the excluded participants are presented in Supplementary Material 2.

**Table 1:** Socio-Demographic characteristics of participants included, by year

	2014 (*n* = 27,633)		2015 (*n* = 33,341)		2016 (*n* = 32,377)		2017 (*n* = 33,218)		2018 (*n* = 34,476)		2019 (*n* = 33,613)		2020 (*n* = 32,422)		2021 (*n* = 32,436)	
	*n*	%	*n*	%	*n*	%	*n*	%	*n*	%	*n*	%	*n*	%	*n*	%
Sex																
Male	12,806	46.3	14,779	48.9	14,127	48.9	14,424	48.5	14,696	48.4	14,301	48.4	14,949	48.5	13,863	48.3
Female	14,827	53.7	18,562	51.1	18,250	51.1	18,794	51.5	19,780	51.6	19,312	51.6	17,473	51.5	18,573	51.7
Age																
15–34	11,022	43.1	16,564	45.3	15,500	44.4	15,913	43.6	16,121	42.7	15,496	42.3	14,259	42.3	15,881	42.9
35–54	9395	33.9	10,886	33.5	10,742	33.8	10,819	34.5	11,692	34.6	11,150	34.8	11,248	34.7	10,901	34.3
55–74	5460	18.0	4680	16.4	4791	16.8	5,030	17.0	5,332	17.7	5405	17.8	5731	17.9	4546	17.9
75 +	1756	5.0	1211	4.9	1344	5.0	1,456	4.9	1,331	5.0	1562	5.1	1184	5.1	1108	5.0
Area																
Urban	10,663	75.1	11,446	65.5	11,113	64.8	11,349	79.3	11,923	80.4	11,842	80.8	11,487	80.7	20,891	80.7
Rural	16,970	24.9	21,895	34.5	21,264	35.2	21,869	20.8	22,553	19.6	21,771	19.2	20,935	19.3	11,545	19.3
Wealth index																
Very low	8151	18.7	9366	26.3	8967	26.4	10,045	18.5	11,019	18.7	10,860	18.5	10,403	18.3	10,636	19.0
Low	6782	19.2	8369	21.2	8464	22.0	8575	20.8	8514	20.6	8540	21.2	8112	20.5	8138	20.4
Middle	5153	19.9	6453	18.3	6362	18.5	6368	21.0	6379	20.9	6056	20.6	6081	21.2	5874	20.8
High	4103	21.0	5087	17.7	5059	17.4	4846	20.3	4909	20.4	4625	19.9	4502	20.2	4563	20.0
Very high	3444	21.2	4066	16.4	3525	15.8	3384	19.4	3655	19.4	3532	19.7	3,324	19.8	3225	19.8
Region																
Coastal	10,675	57.2	13,462	50.8	13,005	49.7	13,382	61.7	13,917	62.7	13,304	63.5	12,775	63.2	12,869	62.2
Highland	11,398	30.4	11,842	33.3	11,472	34.0	11,784	25.9	12,622	25.1	12,496	24.5	12,051	24.6	11,573	24.7
Jungle	5560	12.4	8037	15.8	7900	16.4	8052	12.4	7937	12.2	7813	12.0	7596	12.2	7994	13.2
Civil status																
Married	16,327	57.8	22,592	63.6	21,528	63.2	21,986	62.5	23,111	61.9	22,130	62.0	20,384	59.2	21,049	59.7
Never married	6469	29.4	6281	23.0	6062	22.5	6172	22.4	6152	22.4	6087	22.1	6804	24.6	5820	21.8
Previous	4837	12.8	4468	13.4	4787	14.3	5060	15.1	5213	15.7	5396	16.0	5234	16.2	5567	18.5
Prevalence																
Non-symptoms	20,627	77.0	25,215	76.0	24,432	76.2	25,744	79.5	27,127	79.6	26,010	79.0	25,279	77.6	25,412	77.5
Mild	4789	16.2	5617	16.4	5487	16.4	5127	14.1	5094	14.2	5194	14.2	5147	16.2	4938	15.6
Moderate	1379	4.2	1570	4.7	1499	4.4	1409	4.0	1363	3.9	1468	4.2	1207	3.8	1280	4.3
Severe	838	2.7	939	2.9	959	3.0	938	2.4	892	2.4	941	2.6	789	2.5	806	2.7
Proportion of cases treated^a^																
Mild	310	7.4	352	6.2	354	6.8	303	7.3	314	7.4	287	6.9	295	6.8	297	7.9
Moderate	104	8.8	132	7.5	125	7.9	107	8.0	115	9.5	117	9.7	94	10.1	121	10.2
Severe	104	16.3	109	13.2	106	12.5	88	11.6	94	11.8	107	17.3	86	14.0	85	13.6

In all the analyses, the weighted proportion by complex sampling was used

^a^The values do not add up to 100% since they come from the number of people with that level of depressive symptoms

### Depressive symptomatology and proportions of cases treated

Autocorrelation tests identified that both the burden of depressive symptoms and the proportion of treated cases were autocorrelated (Supplement material 3). Therefore the analyses considered lag(1) to correct for the autocorrelation effect.

**Fig. 1:**
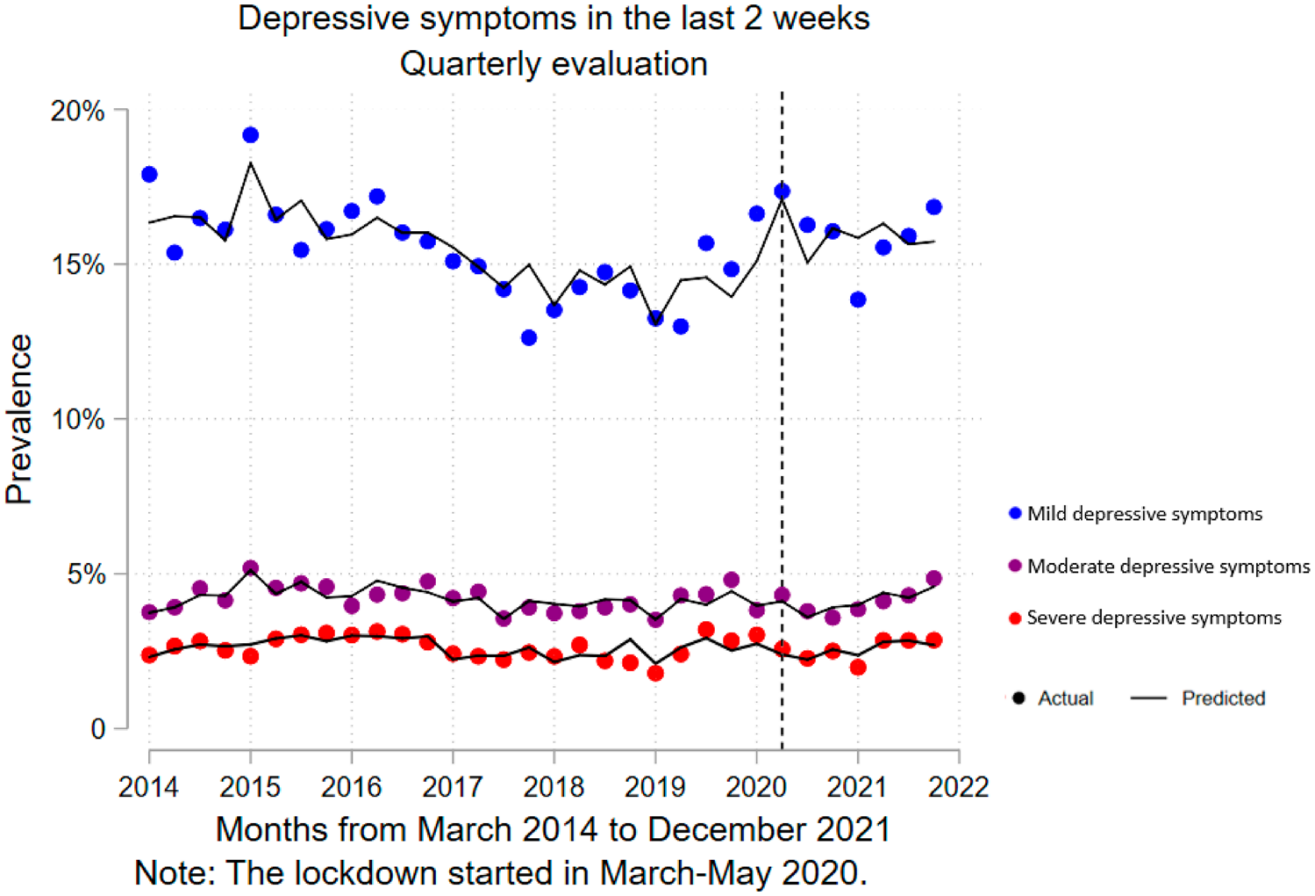
Interrupted time series analysis (quarterly) for depressive symptoms according to level of intensity (adjusted model). Blue color (first dotted line) are mild depressive symptoms. Purple color (second dotted line) are moderate depressive symptoms. Red color (third dotted line) are severe depressive symptoms. Adjusted model by sex, wealth index, and age. In all the analyses, the weighted proportion by complex sampling was used. The first measurement on the dotted line corresponds to June 2020

An average quarterly increase of 0.17% (95% CI 0.03–0.32%) in the prevalence of moderate depressive symptoms was identified after the onset of the COVID-19 pandemic (see Fig. 1), this estimated slope change would correspond approximately to an increase of 1583 new cases of moderate depressive symptoms by each quarter. Similarly, both crude and adjusted models showed an increase of 2.51% (CI 95 0.71–4.30%) and 2.31% (CI 95 − 0.06 to 4.68%), respectively, in mild depressive symptomatology level change. However, the model adjusted for sex, age, and wealth index revealed no slope change of severe depressive symptoms (see Table 2). At a descriptive level, there was an increase in the level of depressive symptoms at all levels of severity immediately after the first lockdown, and then a reduction in prevalence.

**Table 2:** Interrupted time series regression analysis (raw and adjusted model)

	Raw model			Adjusted model^a^		
	Estimated Impact	*p*	[95% Confidence Interval]	Estimated Impact	*p*	[95% Confidence Interval]
Prevalence in the last two weeks						
Mild						
Change in prevalence	2.51	0.008	0.71 to 4.30	2.31	0.055	− 0.06 to 4.68
Change in prevalence trend	0.03	0.887	− 0.39 to 0.44	− 0.07	0.824	− 0.71 to 0.57
Moderate						
Change in prevalence	− 0.23	0.546	− 1.00 to 0.54	− 0.60	0.094	− 1.31 to 0.11
Change in prevalence trend	0.13	0.105	− 0.03 to 0.28	0.17	0.020	0.03 to 0.32
Severe						
Change in prevalence	− 0.22	0.391	− 0.73 to 0.29	− 0.04	0.899	− 0.66 to 0.59
Change in prevalence trend	0.09	0.019	0.02 to 0.17	0.03	0.551	− 0.08 to 0.14
Proportion of cases treated						
Mild						
Change in prevalence	0.26	0.641	− 0.87 to 1.39	− 0.46	0.499	− 1.87 to 0.94
Change in prevalence trend	0.09	0.333	− 0.09 to 0.27	0.46	0.001	0.20 to 0.71
Moderate						
Change in prevalence	1.23	0.300	− 1.15 to 3.60	1.27	0.445	− 2.13 to 4.67
Change in prevalence trend	− 0.43	0.129	− 1.00 to 0.13	− 0.36	0.477	− 1.41 to 0.68
Severe						
Change in prevalence	0.45	0.885	− 5.84 to 6.74	-0.25	0.955	− 9.28 to 8.79
Change in prevalence trend	0.28	0.600	− 0.79 to 1.34	0.07	0.936	− 1.72 to 1.86

^a^Model adjusted by sex, wealth index, and age. In all the analyses, the weighted proportion by complex sampling was used. Change in the prevalence of depressive symptomatology at the beginning of the COVID-19 lockdown (Change in intercept). Change in the trend of prevalence in depressive symptomatology over the time after 1 June 2020 (Change in slope [Interaction]). Estimated Impact (Coefficients × 100). Autocorrelation in lag(1) was considered

**Fig. 2:**
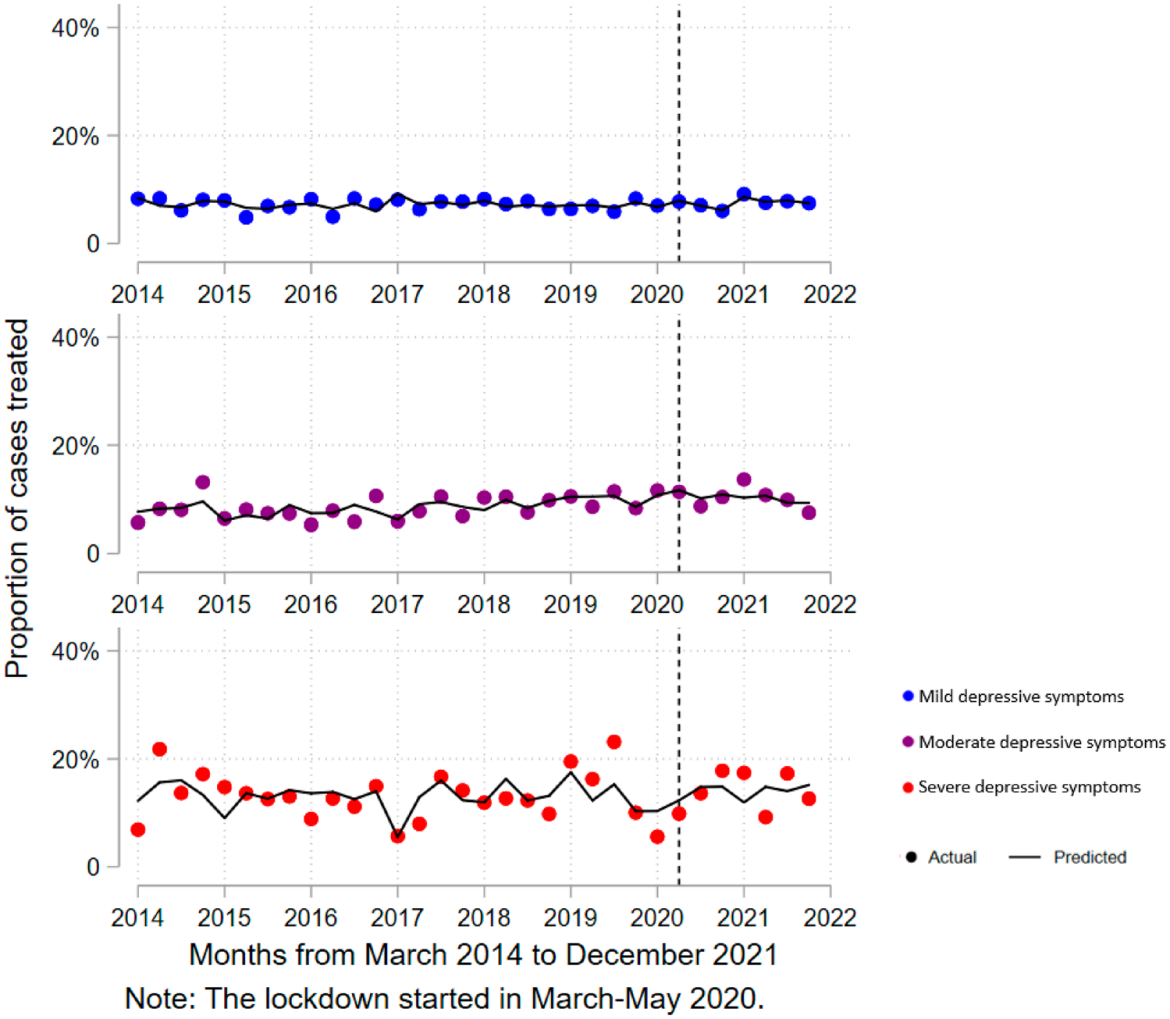
Interrupted time series analysis (quarterly) for proportion of depressive case treated according to level of intensity (adjusted model). Blue color (first dotted line) are mild depressive symptoms. Purple color (second dotted line) are moderate depressive symptoms. Red color (third dotted line) are severe depressive symptoms. Adjusted model by sex, wealth index, and age. In all the analyses, the weighted proportion by complex sampling was used. The first measurement on the dotted line corresponds to June 2020

We identified that the percentage of cases treated for mild depressive symptoms increased quarterly by an average of 0.46% (95% CI 0.20–0.71%) after the onset of the COVID-19 pandemic (see Table 2), this estimated slope change would correspond approximately to an increase of 1242 new cases treated of mild depressive symptoms by each quarter. However, no slope change was found for the percentage of cases treated for moderate or severe depressive symptoms (see Fig. 2).

Estimates from crude models for prevalence of depressive symptoms and proportions of cases treated can be found in Supplement material 4 and 5, respectively. Specific regression estimates can be found in Supplement material 6.

Difference predicted versus counterfactual for the prevalence of mild and moderate depressive symptoms at the end of the second year of the pandemic a difference of 1.89% (95% CI − 1.16 to 4.95; *p* = 0.210) and 0.44% (95% CI − 0.19 to 1.08%; *p* = 0.159), respectively. On the other hand, the proportion of treated cases with mild depressive symptoms was found to increase by 2.28% (95% CI 0.93–3.62%; *p* = 0.002) by the end of the second year of the pandemic (see supplementary material 7).

## Discussion

### Main finding and interpretation

Our study revealed there was a sustained average quarterly increase (slope) of 0.17% in the prevalence of moderate depressive symptoms in Peru after COVID-19 onset, against no significant trend. A plausible explanation for this finding is that moderate depressive symptoms may not have experienced an immediate increase (first quarter after the COVID-19 onset) would be that most cases started with mild depressive symptoms and gradually evolved into moderate depressive symptoms during the following quarters. This is consistent with our findings that identified an immediate increase in the prevalence of mild depressive symptoms, which is no longer observed in 2021.

Our study found an upward trend in the proportion of cases treated for mild depressive symptoms during the pandemic, suggesting that the Peruvian health system has been able to respond in a sustained manner to the mental health needs of the population and reduce the treatment access gap by 2.28% through the last quarter of 2021. There is evidence that the proportion of cases treated was stable before the pandemic [9], suggesting that the health system response during the pandemic may have been responsible for the reduction of the treatment gap. During the pandemic, the Peruvian health system implemented specific guidelines to treat the mental health of persons with COVID-19, health personnel, and victims of violence. In addition, the use of teleconsultations was increased during the first year of the pandemic [23], together with a sustained increase in the annual mental health budget [24]. However, an increase in the proportion of treated cases with moderate or severe symptoms was not identified, so it is still necessary to develop strategies to promote access to treatment in these groups.

### Comparison with other studies

Our results are consistent with a time-series study in England, which identified an increase in depressive symptoms levels in the general population during the early stages of confinement, followed by a rapid decline between the 2nd and 5th week, with symptoms stabilizing between weeks 16 and 20 [25]. Conversely, assuming suicide as an indirect measure of depression, a time series using Peruvian nationwide suicide registry revealed no significant difference of COVID-19 pandemics in monthly numbers of suicides [26].

On the other hand, different results are found in a meta-analytic review, where a considerable increase (7 times greater) in the prevalence of depressive symptoms in the general population is shown. However, high levels of heterogeneity between prevalence estimates in different studies could be attributed to the wide range of measures used [3]. Furthermore, estimates cannot be directly compared since our study estimated absolute effects in the population, whereas the meta-analysis used a ratio-based estimator. In addition, other studies in different continents where validated measurement scales were used also report a small increase in the prevalence of depressive symptomatology [2, 27].

Regarding the prevalence of mild, moderate, and severe levels of depressive symptomatology, our findings show that the prevalence of mild depressive symptomatology decreases slightly faster throughout the confinement compared to the other levels, consistent with the result of another study, where it was found that participants who reported symptoms of low-level depressive symptoms during the first 16 weeks of the national lockdown in England subsequently improved [28]. Particularly in Peru, no other time series studies evaluate depressive symptoms during the pandemic context. However, there are different cross-sectional or cohort studies that report high levels of depressive and anxious symptoms during the pandemic context [29–31].

Although an increase in treated cases with psychiatric symptoms such as moderate and severe depressive symptoms was expected as a result of the pandemic, various barriers to access to diagnosis and treatment persist, including stigma, and others specific to the current situation such as the extension of the confinement to control the spread of the SARS-CoV-2 virus and difficulties in the implementation of technological solutions [32]. The increase in the supply of mental health services through technological tools could partly explain the increase in the proportion of people with mild depressive symptoms who are treated. This is linked to a massive adoption of telehealth services, with a 6000% increase in daily care in the early stages of COVID-19 in Peru, of which approximately 71% was for mental health care [33, 34].

Other studies reported a decrease in the number of psychiatric emergency consultations during the confinement period, compared to other diseases, and depressive disorders presented a lower proportion in cases treated during 2020 compared to 2019. One hypothesis is that patients could be fearful of seeking care due to fear of infecting themselves and their families, but it is still not clear what factors prevented or favored access to treatment [35]. Also, there is evidence of a greater reduction in the total number of patients who attend an outpatient clinic after confinement and in the number of follow-up visits [36].

Finally, people with pre-existing mental health disorders reported an increase in symptoms and limited and poorer access to services since the beginning of the pandemic; evidencing the existence of barriers to treatment accessibility, such as stigma in health institutions [37], inadequate financial support, and an exacerbation of socioeconomic inequalities [38]. In addition, it was found that these access barriers tend to affect people with socioeconomic difficulties more than the general population [36], and subjects in residential treatment centers are more likely to seek psychiatric care compared to people with symptoms who stay at home [35].

### Limitations and strengths

The main strengths of our study are that it performs a representative assessment of the Peruvian population so that our results are generalizable. In addition, our study is the first to use interrupted time series to assess the impact of the COVID-19 pandemic on depressive symptoms in Peru.

Some limitations must be considered when interpreting our results. First, before the onset of the pandemic, our study assesses 25 quarters, but after the lockdown, it only assesses seven quarters. Therefore, having few measurement points after the onset of the lockdown could limit the study’s ability to assess the impact on depressive symptoms in the long or medium term. Second, the study analyzes a time series of data from different participants; therefore, within-subject mental health changes could not be measured, only changes in population estimates. Third, the evaluation of depressive symptomatology was carried out with the PHQ-9 self-report instrument, different from a diagnostic clinical interview; other studies have reported differences in prevalence according to the instrument used. Fourth, different evaluation years use different data collection processes (2014–2019 was face-to-face, and 2020–2021 was virtual and face-to-face), so there could be errors between measurements [25]. Nevertheless, although some evidence suggests data collection methods could be a source of variation regarding prevalence and mean values, other studies point out that means of negative mental health outcomes [26], as depression or anxiety, did not show significant differences comparing telephone and face-to-face interviews [3]. Fifth, the data may not be representative at the quarterly level since in some cases there is only one month within the evaluated quarter. Sixth, our study assumes the assumption of exchangeability between the pre-pandemic period and the post-pandemic period; that is, these are comparable and, therefore, the causal effect can be estimated. In the crude model we assumed unconditional exchangeability, so direct comparison between groups returns an estimation of the impact of COVID-19 pandemic. The change of some characteristics over time could confound the estimate of the effect of interest, thus we adjusted for the composition of the population in terms of sex, wealth index, and age (adjusted model). In this case, we assumed conditional exchangeability, so the impact of COVID-19 could be estimated after conditioning on confounding variables. However, residual confounding remains a potential threat like any observational study. Seventh, there is also the risk of ecological fallacy since we are assuming that the change in prevalence at the district level reflects the change at the individual level. Nonetheless, there is no plausible mechanism by which changes at the population level do not reflect changes at the individual level, so we consider that the risk of bias due to the ecological fallacy is low. Eighth, there may have been classification bias in people who may have been misclassified as having depressive symptoms. The PHQ-9 includes somatic or negative indicators that are frequent in other physical or mental health problems. Therefore, some people with high somatic scores may have been misclassified as having depressive symptoms. However, the number of people misclassified should be low since the PHQ-9 has adequate levels of sensitivity and specificity (sensitivity = 0.86 and specificity = 0.85) [39]. Ninth, our study using quarterly data may limit the ability to account for seasonality. However, due to the sampling methods used in DHS-P, a quarterly aggregation is the minimum time lag allowed to ensure an acceptable variability regarding health assessment (mainly PHQ-9) to calculate robust findings on a nationwide basis. Finally, the 2020 DHS-P had to iterate its data collection procedure, from in-person to telephone interviews due to the COVID-19 lockdown. This measure could be reflected in the under-estimation of the prevalence of main outcomes [40] due to the lack of telephone access of some groups, as participants from rural settings or with less education level [41].

### Implications for public health

Our study found an increase in mild depressive symptoms immediately after the onset of the pandemic, then declining as the months passed, but counterbalanced by a sustainable increase in moderate depressive symptoms of 0.17% quarterly. According to INEI data, between 2020 and 2021 Peru had a population of approximately 24 million inhabitants aged 15 years and older, so a quarterly increase in the population with moderate depressive symptoms could represent a public health problem in the short-medium term. It should be noted that our estimate is at the level of the general population; specific estimates for vulnerable groups such as low-income people, the elderly, or women could estimate a higher risk. It is recommended to develop and strengthen policies for the prevention of mental health problems and the promotion of well-being, to slow down the increase in cases of depressive symptoms. In particular, physical activity-based and school-based interventions have proven to be effective in preventing the onset of mental health problems [42, 43]. We suggest targeting vulnerable groups with these interventions.

The COVID-19 pandemic context had a positive effect on mental health care policies and the adoption of new health technologies in the Peruvian health system. On the one hand, mental health reform was boosted in terms of political will and budget as it became a priority during the pandemic. Similarly, the pandemic increased people's access to telehealth services through the accelerated adoption of the use of information and communication technologies, the relaxation of telemedicine regulation, and an increased budget for its implementation at the national level. The above is reflected in our results regarding the proportion of people with depressive symptoms who received treatment, which showed that there was no significant variation in the trend, and in the case of people with mild symptoms who were treated, there was a small increase. These may be, also, because of increases the number of Community Mental Health Centers to reduce the gaps in mental health treatment in the Peruvian health system; however, it is still too early to assess the effects of the health reform at the population level. The health system could use these data to regulate its health strategies and implement intelligence teams to develop early mental health assessments.

## Conclusions

In Peru, increases in the prevalence of moderate depressive symptoms and the proportion of cases treated with mild depressive symptoms were found after the COVID-19 pandemic. This study can serve as precedent for future research assessing the prevalence of depressive symptoms and the proportion of cases receiving treatment during the pandemic and post-pandemic years.

## Supplementary Information

Below is the link to the electronic supplementary material.

Supplementary file1 (DOCX 208 KB)

## Data Availability

Data are available in a public, open access repository. The database is freely accessible from the website of the National Institute of Statistics of Peru, URL: http://iinei.inei.gob.pe/microdatos/. The information can be obtained by entering the survey query tab and selecting the ENDES data using the health module data. Only cross- sectional information from 2014 to 2021 for the ENDES Health Questionnaire was used.
