# Supplementary material for: Estimated impact of the COVID-19 pandemic on the prevalence and treatment of depressive symptoms in Peru: an interrupted time series analysis in 2014–2021
Source: Soc Psychiatry Psychiatr Epidemiol. 2023 Mar 8;58(9):1375–85. doi: 10.1007/s00127-023-02446-8 (PMC9993377; doi:10.1007/s00127-023-02446-8)
Supplement: Supplementary file 1 — Supplementary file1 (DOCX 208 KB) [file 127_2023_2446_MOESM1_ESM.docx]

**Supplement material 1.** Sampling design and distribution of months, quarters and years evaluated in the DHS-P.

DHS-P has a complex sampling design. In the first stage, a systematic sample of clusters with probability proportional to size was selected defined as a group of houses according to a sampling frame built from the 2007 and 2017 National Census of People and Houses of Peru In the second stage, a random sample of dwellings, balanced by variables of interest, in each selected cluster was selected. In the third stage, only one person per household was randomly selected.

| Quarter | Months / Years | 2014 | 2015 | 2016 | 2017 | 2018 | 2019 | 2020 | 2021 |
| --- | --- | --- | --- | --- | --- | --- | --- | --- | --- |
| 1 | January | No | No | No | No | No | Yes | Yes | Yes |
|  | February | No | No | No | No | Yes | Yes | Yes | Yes |
|  | March | Yes | Yes | Yes | Yes | Yes | Yes | **Yes*** | Yes |
| 2 | April | Yes | Yes | Yes | Yes | Yes | Yes | **Yes*** | Yes |
|  | May | Yes | Yes | Yes | Yes | Yes | Yes | **Yes*** | Yes |
|  | June | Yes | Yes | Yes | Yes | Yes | Yes | Yes | Yes |
| 3 | July | Yes | Yes | Yes | Yes | Yes | Yes | Yes | Yes |
|  | August | Yes | Yes | Yes | Yes | Yes | Yes | Yes | Yes |
|  | September | Yes | Yes | Yes | Yes | Yes | Yes | Yes | Yes |
| 4 | October | Yes | Yes | Yes | Yes | Yes | Yes | Yes | Yes |
|  | November | Yes | Yes | Yes | Yes | Yes | Yes | Yes | Yes |
|  | December | Yes | Yes | Yes | Yes | Yes | Yes | Yes | Yes |

Note: * Months in which the COVID-19 pandemic started and the lockdown occurred.

**Supplement material 2.** Excluded participants.

|  |  | 2014 (n=1160) | | 2015 (n=1130) | | 2016 (n= 1166) | | 2017 (n=881) | | 2018 (n=912) | | 2019 (n=1358) | | 2020 (n=1732) | | 2021 (n=1928) | |
| --- | --- | --- | --- | --- | --- | --- | --- | --- | --- | --- | --- | --- | --- | --- | --- | --- | --- |
|  |  | n | % | n | % | n | % | n | % | n | % | n | % | n | % | n | % |
| Sex | Male | 0 | 0% | 10 | 1% | 0 | 0% | 0 | 0% | 0 | 0% | 0 | 0% | 0 | 0% | 0 | 0% |
|  | Female | 0 | 0% | 19 | 2% | 0 | 0% | 1 | 0% | 0 | 0% | 0 | 0% | 0 | 0% | 0 | 0% |
|  | Missing | 1,160 | 100% | 1,101 | 97% | 1,166 | 100% | 880 | 100% | 912 | 100% | 1,358 | 100% | 1,732 | 100% | 1,928 | 100% |
| Age | 15-34 | 0 | 0% | 16 | 1% | 0 | 0% | 0 | 0% | 0 | 0% | 0 | 0% | 0 | 0% | 0 | 0% |
|  | 35-54 | 0 | 0% | 4 | 0% | 0 | 0% | 0 | 0% | 0 | 0% | 0 | 0% | 0 | 0% | 0 | 0% |
|  | 55-74 | 0 | 0% | 6 | 1% | 0 | 0% | 1 | 0% | 0 | 0% | 0 | 0% | 0 | 0% | 0 | 0% |
|  | 75+ | 0 | 0% | 3 | 0% | 0 | 0% | 0 | 0% | 0 | 0% | 0 | 0% | 0 | 0% | 0 | 0% |
|  | Missing | 1,160 | 100% | 1,101 | 97% | 1,166 | 100% | 880 | 100% | 912 | 100% | 1,358 | 100% | 1,732 | 100% | 1,928 | 100% |
| Area | Rural | 261 | 23% | 296 | 26% | 253 | 22% | 194 | 22% | 175 | 19% | 206 | 15% | 401 | 23% | 415 | 22% |
|  | Urban | 899 | 78% | 834 | 74% | 913 | 78% | 687 | 78% | 737 | 81% | 1,152 | 85% | 1,331 | 77% | 1,513 | 78% |
| Wealth index | Very low | 207 | 18% | 265 | 23% | 214 | 18% | 181 | 21% | 155 | 17% | 212 | 16% | 339 | 20% | 384 | 20% |
|  | Low | 205 | 18% | 208 | 18% | 209 | 18% | 152 | 17% | 162 | 18% | 237 | 17% | 334 | 19% | 327 | 17% |
|  | Middle | 230 | 20% | 198 | 18% | 232 | 20% | 152 | 17% | 199 | 22% | 279 | 21% | 367 | 21% | 400 | 21% |
|  | High | 241 | 21% | 227 | 20% | 258 | 22% | 204 | 23% | 159 | 17% | 316 | 23% | 358 | 21% | 377 | 20% |
|  | Very high | 277 | 24% | 232 | 21% | 253 | 22% | 192 | 22% | 237 | 26% | 314 | 23% | 334 | 19% | 440 | 23% |
| Region | Coastal | 611 | 53% | 494 | 44% | 576 | 49% | 448 | 51% | 499 | 55% | 838 | 62% | 942 | 54% | 1,031 | 53% |
|  | Highlands | 375 | 32% | 390 | 35% | 394 | 34% | 274 | 31% | 277 | 30% | 366 | 27% | 509 | 29% | 613 | 32% |
|  | Jungle | 174 | 15% | 246 | 22% | 196 | 17% | 159 | 18% | 136 | 15% | 154 | 11% | 281 | 16% | 284 | 15% |
| Civil status | Married | 625 | 54% | 718 | 64% | 755 | 65% | 538 | 61% | 560 | 61% | 823 | 61% | 980 | 57% | 1,257 | 65% |
|  | Never married | 412 | 36% | 279 | 25% | 298 | 26% | 240 | 27% | 267 | 29% | 374 | 28% | 541 | 31% | 468 | 24% |
|  | Previous | 123 | 11% | 113 | 10% | 113 | 10% | 103 | 12% | 85 | 9% | 161 | 12% | 211 | 12% | 203 | 11% |
|  | Missing | 0 | 0% | 20 | 2% | 0 | 0% | 0 | 0% | 0 | 0% | 0 | 0% | 0 | 0% | 0 | 0% |

Note: Values were not adjusted for the weighting factor.

**Supplement material 3.** P-values from Cumby-Huizinga test for autocorrelation (raw and adjusted model).

|  |  | Raw Model | | | Adjusted model | | |
| --- | --- | --- | --- | --- | --- | --- | --- |
|  |  | lag | | | lag | | |
|  |  | 1 | 2 | 3 | 1 | 2 | 3 |
| Prevalence in the last two weeks | Mild | *0.0492 | 0.6480 | 0.1675 | 0.9852 | 0.5580 | 0.0671 |
|  | Moderate | 0.2873 | *0.0268 | 0.8792 | 0.2470 | 0.7644 | 0.3919 |
|  | Severe | *0.0018 | 0.5796 | 0.8228 | *0.0365 | 0.3081 | 0.2171 |
| Proportion of cases treated | Mild | 0.0960 | 0.5033 | 0.5421 | 0.0899 | 0.4124 | 0.9029 |
|  | Moderate | *0.0499 | 0.3215 | 0.9215 | 0.0623 | 0.5868 | 0.2229 |
|  | Severe | 0.9666 | 0.6696 | 0.2317 | 0.5035 | 0.6766 | 0.2751 |

Note: *There are some p value < 0.05 then in some cases there is autocorrelation, specifically in lag(1) in most cases. Our interrupted time series models considered autocorrelation in lag(1).

**
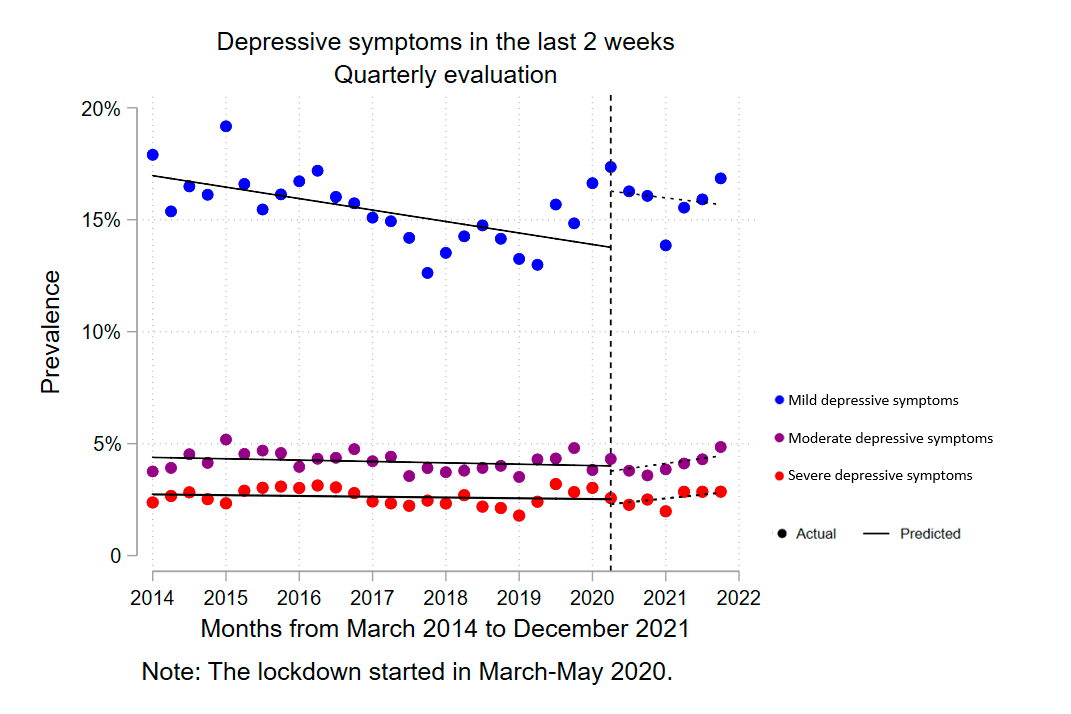
**

**Supplement material 4.** Interrupted time series analysis (quarterly) for depressive symptoms according to level of intensity (raw model).

Note: Blue color (first dotted line) are mild depressive symptoms. Purple color (second dotted line) are moderate depressive symptoms. Red color (third dotted line) are severe depressive symptoms. In all the analyses, the weighted proportion by complex sampling was used. The first measurement on the dotted line corresponds to June 2020.

**
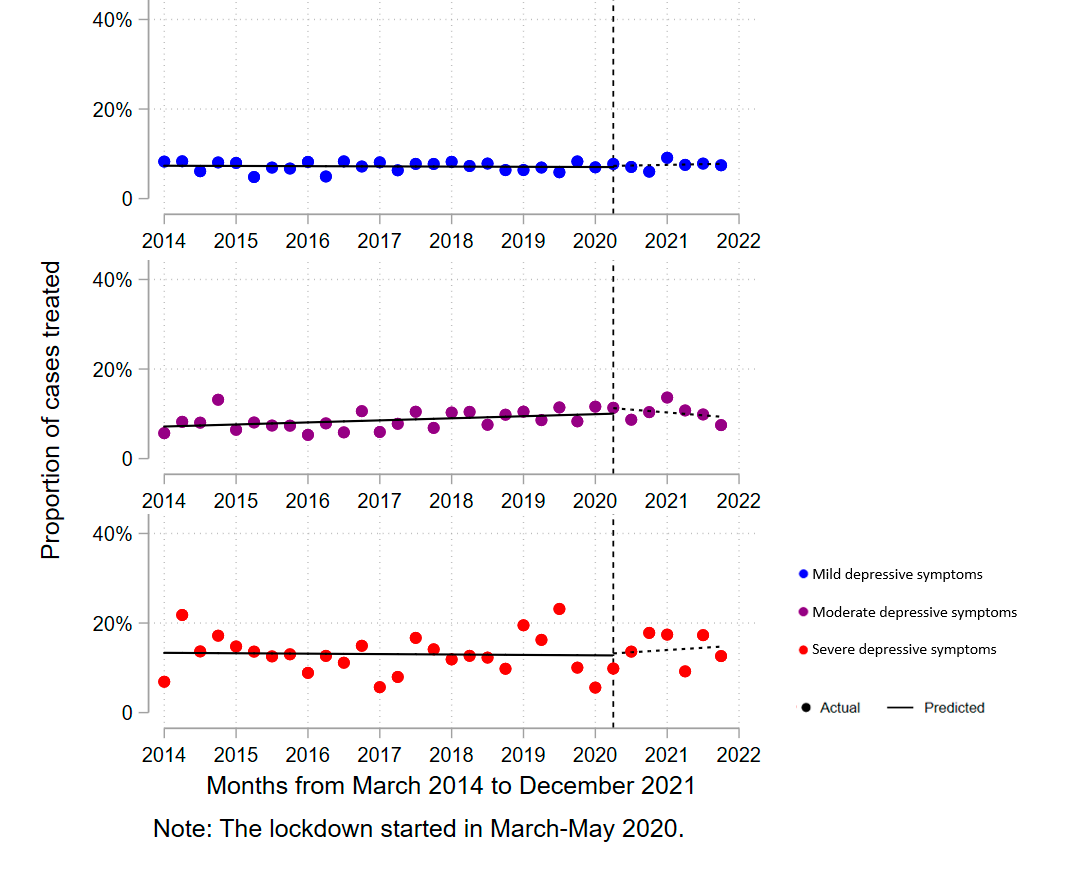
**

**Supplement material 5.** Interrupted time series analysis (quarterly) for proportion of depressive cases treated according to level of intensity (raw model). Note: Blue color (first dotted line) are mild depressive symptoms. Purple color (second dotted line) are moderate depressive symptoms. Red color (third dotted line) are severe depressive symptoms. Adjusted model by sex, wealth index, and age. In all the analyses, the weighted proportion by complex sampling was used. The first measurement on the dotted line corresponds to June 2020.

**Supplement material 6.** Interrupted time series regression analysis (raw and adjusted model) con lag (1).

|  |  |  | Raw model | | Adjusted model* | | |
| --- | --- | --- | --- | --- | --- | --- | --- |
|  |  |  | Coefficients | *p* | Coefficients | *p* | [95% Confidence Interval] |
| Prevalence in the last two weeks | Mild | Intercept | 0.1697 | 0.000 | 0.3941 | 0.402 | -0.5670 to 1.3552 |
|  |  | Pre intervention slope | -0.0013 | 0.008 | -0.0007 | 0.140 | -0.0016 to 0.0002 |
|  |  | Change in intercept | 0.0251 | 0.008 | 0.0231 | 0.055 | -0.0006 to 0.0468 |
|  |  | Change in slope (Interaction) | 0.0003 | 0.887 | -0.0007 | 0.824 | -0.0071 to 0.0057 |
|  |  | Post intervention linear trend | -0.0010 | 0.604 | -0.0014 | 0.642 | -0.0075 to 0.0047 |
|  | Moderate | Intercept | 0.0439 | 0.000 | 0.4774 | 0.478 | 0.2758 to 0.6790 |
|  |  | Pre intervention slope | -0.0002 | 0.302 | 0.0000 | 0.820 | -0.0002 to 0.0002 |
|  |  | Change in intercept | -0.0023 | 0.546 | -0.0060 | 0.094 | -0.0131 to 0.0011 |
|  |  | Change in slope (Interaction) | 0.0013 | 0.105 | **0.0017** | **0.020** | **0.0003 to 0.0032** |
|  |  | Post intervention linear trend | 0.0011 | 0.148 | **0.0018** | **0.018** | **0.0003 to 0.0032** |
|  | Severe | Intercept | 0.0273 | 0.000 | 0.2273 | 0.088 | -0.0367 to 0.4913 |
|  |  | Pre intervention slope | -0.0001 | 0.532 | 0.0001 | 0.505 | -0.0002 to 0.0004 |
|  |  | Change in intercept | -0.0022 | 0.391 | -0.0004 | 0.899 | -0.0066 to 0.0059 |
|  |  | Change in slope (Interaction) | 0.0009 | 0.019 | 0.0003 | 0.551 | -0.0008 to 0.0014 |
|  |  | Post intervention linear trend | 0.0008 | 0.015 | 0.0004 | 0.391 | -0.0006 to 0.0014 |
| Proportion of cases treated | Mild | Intercept | 0.0734 | 0.000 | 0.0275 | 0.934 | -0.6610 to 0.7160 |
|  |  | Pre intervention slope | -0.0001 | 0.652 | **-0.0007** | **0.012** | **-0.0013 to -0.0002** |
|  |  | Change in intercept | 0.0026 | 0.641 | -0.0046 | 0.499 | -0.0187 to 0.0094 |
|  |  | Change in slope (Interaction) | 0.0009 | 0.333 | **0.0046** | **0.001** | **0.0020 to 0.0071** |
|  |  | Post intervention linear trend | 0.0008 | 0.3816 | **0.0038** | **0.004** | **0.0014 to 0.0063** |
|  | Moderate | Intercept | 0.0716 | 0.000 | 0.0005 | 1.000 | -2.7493 to 2.7504 |
|  |  | Pre intervention slope | 0.0011 | 0.025 | 0.0007 | 0.483 | -0.0014 to 0.0028 |
|  |  | Change in intercept | 0.0123 | 0.300 | 0.0127 | 0.445 | -0.0213 to 0.0467 |
|  |  | Change in slope (Interaction) | -0.0043 | 0.129 | -0.0036 | 0.477 | -0.0141 to 0.0068 |
|  |  | Post intervention linear trend | -0.0032 | 0.2528 | -0.0029 | 0.522 | -0.0122 to 0.0064 |
|  | Severe | Intercept | 0.1335 | 0.000 | -2.3146 | 0.219 | -6.1173 to 1.4881 |
|  |  | Pre intervention slope | -0.0002 | 0.883 | 0.0002 | 0.913 | -0.0038 to 0.0043 |
|  |  | Change in intercept | 0.0045 | 0.885 | -0.0025 | 0.955 | -0.0928 to 0.0879 |
|  |  | Change in slope (Interaction) | 0.0028 | 0.600 | 0.0007 | 0.936 | -0.0172 to 0.0186 |
|  |  | Post intervention linear trend | 0.0025 | 0.5936 | 0.0009 | 0.9094 | -0.0156 to 0.0175 |

Note: *Model adjusted by sex, wealth index, and age. In all the analyses, the weighted proportion by complex sampling was used. Intercept: The prevalence of depressive symptomatology at the beginning of the study period. Pre intervention slope: The previous trend of prevalence in depressive symptomatology. Change in intercept: Change in the prevalence of depressive symptomatology at the beginning of the COVID-19 lockdown. Change in slope (Interaction): Change in the trend of prevalence in depressive symptomatology over the time after 1 June 2020. Autocorrelation in lag(1) was considered.

**Supplement material 7.** Difference predicted versus counterfactual in the last quarter for 2020 and 2021.

|  |  | Raw model | | | | Adjusted model* | | | |
| --- | --- | --- | --- | --- | --- | --- | --- | --- | --- |
|  |  | Octuber-December 2020 | | Octuber-December 2021 | | Octuber-December 2020 | | Octuber-December 2021 | |
|  |  | Estimated Impact | p | Estimated Impact | p | Estimated Impact | p | Estimated Impact | p |
| Prevalence in the last two weeks | Mild | 2.56 (0.8 to 4.32) | 0.006 | 2.68 (0.04 to 5.32) | 0.047 | **2.17 (0.28 to 4.06)** | **0.026** | 1.89 (-1.16 to 4.95) | 0.210 |
|  | Moderate | 0.03 (-0.59 to 0.64) | 0.930 | 0.54 (-0.19 to 1.27) | 0.140 | -0.25 (-0.80 to 0.30) | 0.355 | 0.44 (-0.19 to 1.08) | 0.159 |
|  | Severe | -0.03 (-0.57 to 0.51) | 0.906 | 0.34 (-0.36 to 1.04) | 0.325 | 0.02 (-0.58 to 0.62) | 0.935 | 0.15 (-0.61 to 0.91) | 0.688 |
| Proportion of cases treated | Mild | 0.44 (-0.56 to 1.43) | 0.376 | 0.79 (-0.3 to 1.88) | 0.150 | 0.45 (-0.73 to 1.63) | 0.436 | **2.28 (0.93 to 3.62)** | **0.002** |
|  | Moderate | 0.36 (-1.57 to 2.29) | 0.705 | -1.37 (-4.13 to 1.38) | 0.316 | 0.55 (-2.24 to 3.33) | 0.687 | -0.90 (-6.03 to 4.22) | 0.717 |
|  | Severe | 1.00 (-5.15 to 7.15) | 0.742 | 2.10 (-5.75 to 9.95) | 0.588 | -0.11 (-7.75 to 7.53) | 0.976 | 0.17 (-9.26 to 9.60) | 0.970 |

Note: *Model adjusted by sex, wealth index, and age. In all the analyses, the weighted proportion by complex sampling was used. Estimated Impact (Coefficients x 100). Autocorrelation in lag(1) was considered.
